# Willingness to pay for social health insurance and its associated factors among public servants in Addis Ababa, Ethiopia: a cross-sectional study

**DOI:** 10.1186/s12913-022-08304-8

**Published:** 2022-07-13

**Authors:** Melkamu Ayalew Kokebie, Ziyad Ahmed Abdo, Shikur Mohamed, Belayneh Leulseged

**Affiliations:** 1 Department of Maternal, Child Health and Nutrition, Ethiopian Ministry of Health, Addis Ababa, Ethiopia; 2 Department of Hygiene and Environmental Health, Ethiopian Ministry of Health, Addis Ababa, Ethiopia; 3 School of Public Health, St. Paul’s Hospital Millennium Medical College, Addis Ababa, Ethiopia

**Keywords:** Willingness to pay, Social health insurance, Associated factors, Addis Ababa

## Abstract

**Background:**

As a means of establishing a sustained and fair health care financing system, Ethiopia has planned and ratified a legal framework to introduce a social health insurance program for employees of the formal sector to protect them against financial and health burdens. However, the implementation has been delayed due to the resistance of public servants to pay the proposed premium. The aim of this study was to estimate the magnitude of willingness to pay the proposed amount of premium set by the government for the social health insurance program and the factors associated with it among public servants in Addis Ababa, Ethiopia.

**Methods:**

An institution-based cross-sectional study design was used to conduct the study. Multistage sampling was employed to select a total of 503 from 12 randomly selected public sectors. Data were collected using pretested, interviewer-administered structured questionnaires. A contingent valuation method with an iterative bidding game was used to elicit willingness to pay. Finally, logistic regression analysis was used to identify independent predictors of willingness to pay. Statistical significance was considered at *P* < 0.05 with adjusted odds ratios calculated at 95% CIs.

**Results:**

Only 35.4% were willing to pay the proposed premium (3% of their monthly salary). Those who had children from 6–18 years old (AOR = 3.252; 95% CI: 1.15, 9.22), had a history of unaffordable health service costs during the last 12 months (AOR = 9.631; 95% CI: 4.12, 22.52), and had prior information about the social health insurance program (AOR = 11.011, 95% CI. 3.735–32.462) were more likely to pay for the proposed social health insurance program compared to their counterparts.

**Conclusion:**

The willingness to pay the proposed amount premium for social health insurance among public servants in Addis Ababa was very low that implies the implementation will be challenging. Thus, the government of Ethiopia should consider reviewing the amount of premium contributions expected from employees before implementing the social health insurance scheme.

## Introduction

Revenue raising, pooling and purchasing of health services are the key functions of the health financing system [1]. The health system should have a good health financing system that ensures that people use health services without financial catastrophe or impoverishment [2, 3]. This can be done by designing insurance systems [4]. The social health insurance (SHI) scheme is one of the possible organizational mechanisms for raising and pooling funds to finance health services, along with tax financing, private health insurance, community-based health insurance, and others [4, 5].

Pooling of prepaid funds has received due attention in the health financing mechanisms of the health system framework designed by the World Health Organization (WHO) [6]. The system pools both the health risks of the people on the one hand and the contributions of employees, employers and the government on the other [7]. Thus, it protects people against financial and health burdens and is a relatively fair method of financing health care [8, 9]. To achieve universal health coverage (UHC), social health insurance plays a crucial role without incurring financial hardship [8, 10, 11].

Developed countries often use SHI to mobilize funds and pool risks, but low- and middle-income countries rarely use this approach [11]. In Ethiopia, the strategic health care financing system is a recent practice that began in the 1990s [12], following the 1993 health policy [13]. Health insurance was one of the financing mechanisms of the 1998 health care financing strategy (HCFS) and received more attention in the 2017 revised strategy [14]. According to the 2018 Ethiopian Ministry of Health annual report, only 38% of the eligible population joined the Community health insurance program [15]. Similarly, although legal frameworks were developed and many preparatory activities have been made to implement the Social Health insurance scheme [16, 17] in which government employees shall contribute 3% of their gross salary [17], Ethiopia is still at the introduction stage of SHI [16–19]. One of the main reasons for repeatedly postponing the implementation of the SHI scheme in Ethiopia was public servant’s resistance to pay the proposed amount of premium [12, 20].

Despite the fact that there is no national study on Willingness to pay (WTP) for SHI in Ethiopia, and most of them were conducted in some parts of the country on few type of public servants that made it to generalized, evidence from those studies indicated that public servants willing to pay for SHI varied from place to place [2, 21–23]. For example, according to a study done on public servants in St. Paul’s hospital, Addis Ababa, showed that only 17% were willing to pay the proposed amount of premium [2]. In contrast to this, a study in Northeast Ethiopia showed that 85.3% of public servants were willing to pay and join SHI. However, a focused group discussion in this similar study showed that public servants were agreed to contribute approximately 2% of their gross salary by giving their reasons of low salary, very high cost of living, and burden of other deductions from their salary that implied the need to reduce the proposed premium [22].

The government of Ethiopia has implemented many awareness and demand creation activities for SHI in the country including Addis Ababa. However, the current status of willingness to pay the proposed amount of premium was not well known in Addis Ababa, central Ethiopia. Therefore, this study was conducted to assess the current status of willingness to pay for the proposed SHI scheme and its associated factors among the general public servants in Addis Ababa, Ethiopia.

## Methods

### Study design and setting

This study employed an institution-based cross-sectional design and was conducted from February 24-June 17/2020. The study was conducted among public servants in Addis Ababa, which is the capital city of Ethiopia and has a population of approximately 4.8 million [24]. Administratively, the city has 11 sub Cities and 116 Districts. According to the 2019 report, there were approximately 85 registered types of public institutions that might have its own lower level organizational structures, and 115,398 public servants who were permanently employed at different levels of the city administration [25].

### Population

All employees working in 85 registered public institutions in the Addis Ababa city administration were considered as the source population. The study participants were randomly selected public servants from the sampled public institutions. Public servants who were permanently employed and present during the study period were included in the study. Public servants who were severely ill or could not listen and speak were excluded from the study.

### Sample size and sampling procedures

The required sample size for the first objective of this study was determined by using a single population proportion formula with the following assumptions: 95% confidence level, 5% margin of error and 53% proportion public servants willing to willingness to pay for the proposed SHI, taken from a previous study done in South Ethiopia [26].

$$n=\;\frac{\left(Z_{\displaystyle\frac a2}\right)^2\;\ast p\;\left(1-p\right)}{d^2}$$

Accordingly, this yields a maximum sample size of 383. Adding a 10% nonresponse rate and a design effect of 1.2, the final sample size required for the study was 506.

Sample size for the second objective was calculated by using EPI Info version 7, and using factors associated with WTP. Studies in Ethiopia found that WTP for social health insurance were associated with income, educational status, information status on SHI, health status, quality of health service and affordability of health service. The sample size was calculated by using each listed factors, and unfortunately the sample size we found in each of them was less compared to the sample size calculated for the first objective.

A multistage sampling system was used to select study participants. Initially, 12 public sectors were randomly selected from all 85 public sectors identified in Addis Ababa. In the next stage, offices, directorates or units were selected randomly from the selected public institutions. Finally, lists of public servants were obtained from the human resource department, and participants were selected from each office, directorate or unit proportionally depending on the size of the public institutions using a systematic random sampling technique.

### Variables

#### Dependent variables

Willingness to pay the proposed amount premium for the SHI.

#### Independent variables

Socio-demographic variables (age, sex, educational status, income, marital status, family size, presence of under five children, presence of children from 6–18 years old), level of awareness of social health insurance, health status and affordability of health service costs.

### Data collection tool and quality control

Data were collected using an interviewer-administered structured questionnaire adapted from previous studies. The questionnaire was first prepared in English and then translated to Amharic. A pretest was performed among 5% of the sample size among public servants working in public institutions that were not included in the study. Data were collected by six BSc health science professionals and supervised by two experienced public health specialists. Data collectors and supervisors were trained for two days on procedures and made familiar with the questionnaire.

### Data collection procedure

In measuring WTP using contingent valuation and bidding games, first, the hypothetical market was described to respondents, and a series of questions were asked. In this study, a social health insurance scheme proposed by the government of Ethiopia was taken as a case scenario. First, information was provided on general concepts of health insurance, particularly about the proposed social health insurance scheme. Then, participants were asked for their willingness to pay 3% of their monthly salary for the proposed SHI scheme. The amount of the first bid was purposively selected to assess participants’ willingness to pay for the proposed amount of premium (3% of gross salary) for the planned SHI scheme in Ethiopia. Depending upon the response, the respondents were then asked if they would be willing to pay the next higher or lower amount. If a participant responded ‘yes’ for 3%, the next higher bid (4%) was offered, and if a participant responded “No” for 3%, the next lower bid (2%) was offered. This bidding game was continued until a participant switched his or her response from inclusion or exclusion.

### Operational definitions

- Public institution: All types registered institutions that are directly responsible to report to Addis Ababa City administration that might also have organizational structures to the lower administration level
- Proposed amount of premium: 3% of public servants’ gross salary.
- Willingness to pay for the proposed premium: Willingness to pay 3% of gross monthly salary that was set by the government for the proposed SHI Scheme in Ethiopia

### Data management and analysis

Data was coded and entered into Epi Info version 7 and transferred into SPSS version 21 for analysis. Descriptive statistics, such as frequency tables, were used to present the results. Associations between independent variables and willingness to pay were assessed using both bivariate and multivariable logistic regression analyses. Independent variables with *p* values < 0.25 were entered into multivariable logistic regression to assess the effect of each independent variable when controlling for all other potential confounders. Adjusted odds ratios (AORs) and 95% confidence intervals were estimated, and a *p* value < 0.05 was considered significant.

## Results

### Socio-demographic characteristics of respondents

Of the 506 randomly selected respondents, 503 (99.4%) agreed to participate. Among the participants, more than half (56.3%) were males. The mean age of the respondents was 33.22 (SD ± 7.724), with a range between 21 and 56 years. More than half (53.1%) of the respondents were married, and the average family size of study participants was 2.89 (SD ± 1.690). The majority (74.2%) of respondents had a first degree or above educational level (See Table 1).

**Table 1:** Socio-demographic characteristics of public servants in Addis Ababa City Administration, Central Ethiopia, 2020

Variables	Response (*n* = 503)	Frequency	Percent
Sex	Male	283	56.3
	Female	220	43.7
Age(years)	20–24	46	9.1
	25–29	157	31.2
	30–34	109	21.7
	35–39	77	15.3
	> 39	114	22.7
Religion	Orthodox	328	65.2
	Protestant	97	19.3
	Muslim	63	12.5
	Others^a^	15	3.0
Marital status	Currently Single	255	50.7
	Married	248	49.3
Educational status	Certificate and below	45	8.9
	Diploma holder	85	16.9
	First degree and above	373	74.2
Respondent’s monthly net Monthly income(ETB)	< 3145	121	24.1
	3146–3911	51	10.1
	3912–4725	82	16.3
	> 4725	249	49.5
Family size	1 person	162	32.2
	2–4 persons	253	50.3
	5 and above persons	88	17.5
Presence of under 5 years children	Yes	167	33.2
	No	336	66.8
Presence of Children from 6–18 years old	Yes	144	28.6
	No	359	71.4

Others^a^ = Catholic, Wakefeta, 7^th^ day Adventist

### Health and health care-related characteristics of respondents

Among the study participants, 103 (20.5%) had chronic disease, and 208 (41.4%) experienced at least one episode of any other acute illness in the last 12 months. Among participants who had experienced illness, 93.8% sought treatment for their recent episodes. Out-of-pocket health expenditure (86.7%) was the main means of paying at a point of getting health service. More than half (51.8%) experienced unaffordable health service costs. About one third (36.9%) received their health service from public health facilities, and only 15.9% of them were satisfied with the service quality (See Table 2).

**Table 2:** Health and health care-related characteristics among public servants in the Addis Ababa City Administration, Central Ethiopia, 2020

Variables	Response	Frequency	Percent
Presence of chronic illness among family members (*n* = 503)	Yes	103	20.5
	No	400	79.5
Presence of any illness during the last 12 months(*n* = 503)	Yes	208	41.4
	No	295	58.6
Seeking treatment for the recent episode of illness (*n* = 208)	Yes	195	93.8
	No	13	6.3
Place of treatment sought(*n* = 195)	Public health facility	71	36.4
	Local drug vender	64	32.8
	Private Heath Facility	34	17.4
	Traditional healer	26	13.3
Source of health expenditure(*n* = 195)	Government (Free)	8	4.1
	Self (Out Of Pocket)	176	90.2
	Family	19	9.7
Affordability of health care costs (*n* = 195)	Affordable	94	48.2
	Unaffordable	101	51.8
Satisfaction with quality of services (*n* = 195)	Satisfied	31	15.9
	Neutral	73	37.4
	Dissatisfied	91	46.7
Satisfaction with the cost of health service (*n* = 195)	Satisfied	33	17.0
	Neutral	40	20.5
	Dissatisfied	122	62.9

### Awareness of social health insurance

The study revealed that 70% of the respondents had ever heard about SHI, and mass media was the main source of information for more than half (54.5%) of them. Among those participants who had ever heard about social health insurance, 94% believed that social health insurance has benefits (See Table 3).

**Table 3:** Awareness of social health insurance schemes among public servants in the Addis Ababa City Administration, Central Ethiopia, 2020

Descriptions	Response	Frequency	Percent
Ever heard of social health insurance scheme(*n* = 503)	Yes	352	70.0
	No	151	30.0
Source of information for SHI scheme(*n* = 352)*	Mass Media	211	59.9
	Friends	80	23.5
	Insurance Agency	68	19.7
	Working Institution	32	9.3
	Family	16	4.7
Believe that SHI has benefits (*n* = 352)	Yes	331	94.0
	No	21	6.0
Benefit of SHI scheme(n = 331)^a^	Prevent from unexpected health expenditure	240	72.5
	Help others who can’t afford their medical cost	157	47.4
	Have timely care in times of illness	73	22.1
	Improve health services quality	46	14.1
Information on the source of finance for SHI(*n* = 352)^a^	Members’ contribution	287	81.5
	Employers’ contribution	51	14.5
	Investment income	51	14.5

^a^Multiple response is possible

### Willingness to pay the proposed premium

The present study indicated that only 178 (35.4%) study participants had a willingness to pay for the proposed SHI premium (3% of their gross monthly salary). Out of those who were willing to pay the starting bid, 69 (38.8%) respondents also had a willingness to pay for the next higher bid. Out of the 325 (64.6%) participants who responded did not have a willingness to pay the starting bid, 79 (24.3%) had a willingness to pay for the next lower bid. The overall estimated mean WTP for the proposed SHI was 2.5% (95% CI 2.4–2.6) of their gross monthly salary. More than half (53.4%) were willing to pay less than 3% of their gross salary (See Table 4).

**Table 4:** Willingness to pay for social health insurance among public servants in Addis Ababa City Administration, Central Ethiopia, 2020

Variable	Responses	Frequency	Percent
Willingness to contribute for SHI (*n* = 503)	Yes	382	75.9
	No^a^	121	24.1
WTP the proposed amount of premium (3% of gross monthly salary)	Yes	178	35.4
	No	325	64.6
WTP below 3% of gross salary(*n* = 382)	Yes	204	53.4
	No	178	46.6
WTP above 3% of gross salary(*n* = 382)	Yes	87	22.8
	No	295	77.2

^a^Participants who were not willing to contribute at all

### Factors associated with willingness to pay for the proposed SHI scheme

After multivariable logistic regression analysis, presence of children from 6–18 years old, who experienced unaffordable health service costs, and who had ever heard of the social health insurance scheme were significantly associated with WTP for SHI.

Participants who had family members from 6–18 years of age were 3 times more likely to be willing to pay for the proposed SHI scheme compared to those who had not (AOR = 3.252, 95% CI. 1.147–9.219).

It was also found that participants who experienced unaffordable health service costs were 9 times more willing to pay for the proposed SHI scheme than those who experienced affordable health service costs (AOR = 9.631, 95% CI. 4.118–22.525). In addition, participants who had ever heard about the health insurance scheme were 11 times more likely to be willing to pay than those who had never heard (AOR = 11.011, 95% CI. 3.735–32.462 (See Table 5).

**Table 5:** Factors associated with willingness to pay for social health insurance schemes among public servants in Addis Ababa, Central Ethiopia, 2020

Variables	Response	WTP for SHI		COR (95% CI)	AOR(95% CI)
		**Yes** **No. (%)**	**No** **No. (%)**		
Age of respondents	20–24	36 ( 78.26)	10 ( 21.74)	1	1
	25–29	118( 75.16)	39( 24.84)	1.19(0.54–2.62)	0.43(0.79–2.32)
	30–34	66( 60.55)	43( 39.45)	2.34(1.05–5.22)^a^	0.17(0.024–1.29)
	35–39	41( 53.25)	36( 46.75)	3.16(1.38–7.26)^b^	0.36(0.05–2.72)
	> 39	64( 56.14)	50(43.86)	2.81(1.27–6.21)^a^	0.42(0.05–16.19)
Educational status	Diploma and below	99(76.15)	31( 23.85)	1	1
	Degree and above	226( 60.59)	147(39.41)	2.08(1.32–3.27)^b^	3.10(0.84–22.44)
Marital status	Currently Single	185( 72.55)	70( 27.45)	1	1
	Married	140( 56.45)	108( 43.55)	2.04(1.41–2.96)	1.40(0.39–5.02)
Income(ETB)	< 3145	89(73.55)	32( 26.45)	1	1
	3146–3911	31( 60.78)	20(39.22)	1.79(0.90–3.585)	0.26(0.04–1.83)
	3912–4725	61( 74.39)	21(25.61)	0.96(0.51–1.82)	0.96(0.18–5.13)
	> 4725	144( 57.83)	105( 42.17)	2.03(1.26–3.26)^b^	0.26(0.05–1.42)
Family size	1person	125(77.16)	37(22.84)	1	1
	2–4 persons	155(61.26)	98(38.74)	2.14(1.37–3.33)^b^	0.89(0.23–3.48)
	> = 5 persons	45(51.14)	43(48.86)	3.23(1.85–5.63)^b^	0.57(0.10–3.23)
Presence of under 5 years children	Yes	67( 46.53)	77(53.47)	2.84(1.97–4.38)	1.73(0.73–4.12)
	No	240(71.43)	96(28.57)	1	1
Presence of Children from 6–18 years old	Yes	67(46.53)	77(53.47)	2.84(1.97–4.38)^b^	3.25(1.15–9.22)^a^
	No	270(67.5)	130(32.5)	1	1
Presence of chronic illness among family members	Yes	55(53.40)	48( 46.6)	1.82(1.82–2.64)^b^	2.68(1.00–7.19)^a^
	No	270( 67.5)	130(32.5)	1	1
Affordability of health care	Affordable	70( 74.47)	24( 25.53)	1	1
	Unaffordable	36(35.64)	65(64.36)	5.27(2.84–9.76)	2.68(1.00–7.19)
Ever heard of social health insurance scheme	Yes	191(55.20)	155(44.80)	4.73(2.89–7.72)	11.01(3.73–32.46)^b^
	No	134(85.35)	23(14.65)	1	1

^a^Significant at *P* < 0.05,^b^ Significant at *P* < 0.01, *AOR* Adjusted odds ratio, *COR* Crude odds ratio, *CI* Confidence interval

## Discussion

The establishment and implementation of a fair and resilient health care financing system requires local context studies and recommendations. As part of future evidence, this study aimed to assess the willingness to pay the amount of premium for the proposed social health insurance and the factors associated with it among public servants working in Addis Ababa, Ethiopia.

In the present study, only 35.4% of respondents were WTP the amount of premium for the proposed SHI which is much higher than the findings of a study performed in St. Pauls’ Hospital Millennium Medical College, Addis Ababa Ethiopia, which found that 17% of public servants were WTP for the proposed SHI scheme [2]. This difference may be due to the difference in the study population. Participants in the St. Paul’s Hospital Millennium Medical College study included only health care professionals (HCPs). The main explanation for the lower prevalence of WTP for SHI among HCP in this study is that all government hospitals in Addis Ababa provide free health services for their employees. As a result, HCPs rely on the free health service rather than using SHI. Health professionals have an interest in obtaining free health services. The reason behind this interest was that health workers believe that they should not pay for health services that they are providing for others or that the government should cover their expenses for health care.

In contrast to this, there were also evidences indicating that public servants could be willing to pay more than a premium set by the government. A study conducted in North East Ethiopia, Mekele, in 2017 revealed that WTP for SHI scheme was 3.6% [27]. This showed that WTP in Mekele was higher than both of the proposed premium and the WTP in the current study. Another study in Iran in 2014 also showed that more than half (56.6%) with willing to pay the proposed premium and the average WTP for SHI per person per month was more than the premium for the SHI in Iran which consistently showed that public servants also can be willing to pay more than the premium set by the government [28]. This difference might be due to the difference in socio demographic characteristics, and methodology. Mekele and Addis Ababa are two different cities with different characteristics. Addis Ababa is the capital city of the country with higher cost of living. In addition, the study in Mekele did not used the actual proposed premium(3%) as a first bid while our study used the proposed premium as the first bid for their willingness to pay. This showed that the difference might be due to methodology.

The current study result shows that the overall estimated mean of WTP for SHI was 2.5% of participants’ gross monthly salary, which is less than the amount of premium declared by the Ethiopian government [18]. This is higher than the figure obtained from a previous study performed at the initial stage of SHI introduction among public servants in Addis Ababa which showed that the average willingness of contribution was 1.52% of their gross monthly salary [23]. This improvement may be due to awareness and demand creation efforts made by the government, differences in time of study and/or changes in socioeconomic characteristics of the study participants. However, the finding of this study is lower than the finding of a study conducted in northeastern Ethiopia, Mekele, in 2017, which found a 3.6% WTP for the SHI scheme [27].

Our study found that out-of-pocket (OOP) health expenditure was participants’ main means (86.7%) of payment for their health service costs. This finding is supported by a previous study performed in the southern part of Ethiopia in 2014, which found that 87.7% of the respondents used OOP expenditures [26]. This is also consistent with the report from the Ethiopian Health Account (EHA) in 2016/17. The report of the EHA shows that OOP health expenditure was one of the dominant sources of health care finance in Ethiopia [29]. Depending on a health system dominated by OOP health expenditure contradicts the goal of universal health coverage (UHC). To achieve the goals of UHC, a health system requires financing systems that enable health services without incurring financial hardship at a time of obtaining health services [10].

Participants who had family members aged 6–18 years were more likely to have WTP for the proposed SHI scheme than those who had not. The possible reason might be that those participants who had children from 6–18 years old may believe that the proposed SHI scheme is favoring them over the others. In the current Ethiopian SHI scheme, children above 18 years are not entitled to their parent’s health insurance benefits. The finding of this study is supported by a previous study performed in Mekele in 2017 that reported participants were resisting that eligibility should not be based on age alone but also on their capacity to generate income [27].

Study participants who had ever heard about health insurance schemes prior to this study were more willing to pay than those who had never heard. This finding is consistent with a study performed in South Ethiopia [26] and Vietnam in 2014 [30]. This could be explained by employees who ever heard about SHI being more likely to be aware of the benefits of having health insurance.

Regarding awareness and demand creation, the government of Ethiopia reported that many efforts have been made [15]. However, this study revealed that nearly one-third of public servants (30%) were not ever heard about SHI. As a country that is going to implement SHI, having such a significant portion of public servants who had not heard about SHI will have a negative effect on the implementation of the SHI scheme.

Study participants who had experienced unaffordable health service costs were more willing to pay for the proposed SHI scheme than those who did not experience unaffordable health service costs. This finding is similar to a previous study performed in Wolaita Sodo Town, South Ethiopia [26].

### Strength and limitations of the study

This study was one of the few studies done on the proposed SHI scheme, characterizing the public servants in Addis Ababa, covering more public institutions, employing random sampling, pretesting tools, making statistical adjustments of design effect may ensure validity and reliability of the data.

Study participants were asked their willingness to pay without having previous experiences with SHI. Due to this, they may have ambitious expectations or may not take it as a serious plan going to be realized soon. Due to this, their decision may deviate from the decision they made if they were put themselves in the real situation. Moreover, the starting point for the biding game was decided to be 3% of gross salary (proposed by the government), which might affect participants’ first bid choice decision. This first choice bias may influence participants’ last bid of WTP for SHI.

## Conclusions

The willingness to pay the proposed amount premium for social health insurance among public servants in Addis Ababa was very low that implies the implementation will be challenging. Willingness to pay for SHI was more likely among those participants who had children from 6–18 years old, had a history of unaffordable health service costs and had prior information about the SHI scheme, which were significant determinants of WTP. The government of Ethiopia should consider the current level of WTP and address all related factors prior to implementing SHI in the country. All policy makers and implementers under the government of Ethiopia can use the identified associated factors as potential areas of intervention to introduce accepted and sustainable SHI in the country. Further efforts should be made to create awareness and increase demand for SHI. A comprehensive national assessment should be conducted to understand the overall images of the country on employees’ WTP for SHI schemes. 

## Data Availability

Most of the datasets generated and analyzed during the current study are included in the manuscript, hence, for those who need the underling row data, they can get from corresponding author on reasonable request.

## Abbreviations

CBHI: Community-Based Health Insurance
CVM: Contingent Valuation Method
EHA: Ethiopian Health Account
HCFS: Health Care Financing Strategy
OOP: Out of Pocket
REC: Research and Ethics Committee
SHI: Social Health Insurance
SPSS: Statistical Package for Social Science
UHC: Universal Health Coverage
WHO: World Health Organization
WTP: Willingness to Pay

## References

[1] WHO. Health Systems Governance & Financing. Health Financing Guidline No 1:Health financing country diagnostic: a foundation for national strategy development. Vol 200, Autonomic Neuroscience: Basic and Clinical. 2016. Report No.: 1. https://www.who.int/health_financing/documents/country-diagnostic/en/. [Last accessed on 2022 Jan 2].

[2] Lasebew Y, Mamuye Y, Abdelmenan S (2017). Willingness to Pay for the Newly Proposed Social Health Insurance among Health Workers at St. Paul’s Hospital Millennium Medical College, Addis Ababa, Ethiopia. Int J Heal Econ Policy.

[3] Kofoworola Y, Id O, Akomolafe B, Ohiri K (2019). Factors influencing willingness and ability to pay for social health insurance in Nigeria. PLoS ONE.

[4] WHO. The world health report. Health systems improving performance. 2000. https://www.who.int/whr/2010/en/. [Last accessed on 2021 Jan 15].

[5] Ole Doetinchem GC and DE. Thinking of introducing social health insurance ? Ten questions health system financing. the pathto universal coverage. Geneva; 2010. https://www.who.int/healthsystems/topics/financing/healthreport/26_10Q.pdf?ua=1.[Last accessed on 2021 Feb 02].

[6] WHO. Everybody’s business: strengthening health systems to improve health outcomes: WHO’s Framwork for Action. Geneva: WHO press; 2007. https://www.who.int/healthsystems/strategy/everybodys_business.pdf. [Last accessed on 2021 Dec 15].

[7] Le Gall-Ely M (2009). Definition, Measurement and Determinants of the Consumer’s Willingness to Pay: A Critical Synthesis and Directions for Further Research. Rech Appl en Mark (English Ed.

[8] WHO. Social Health Insurance:Report of a Regional Expert Group Meeting. New Delhi; 2003. https://apps.who.int/iris/handle/10665/206364. [Last accessed on 2021 Dec 11].

[9] Chiwire P, Evers SM, Mahomed H, Hiligsmann M (2021). Willingness to pay for primary health care at public facilities in the Western Cape Province, Cape Town. South Africa J Med Econ.

[10] WHO. The World Health Report. Health System Financing. 2010. https://www.who.int/whr/2010/en/. [Last accessed on 2021 Dec 15].

[11] Hsiao WC, Shaw RP, Fraker A, Jowett M. Social Health Insurance for Developing Nations - World Bank Institute development studies. Washington, D. C; 2007. 1–184 bl. https://openknowledge.worldbank.org/handle/10986/6860. [Last accessed on 2022 Feb 04]

[12] Alebachew A, Yusuf Y, Mann C, Berman P. Ethiopia ’ s Progress in Health Financing and the Contribution of the 1998. Health Care and Financing Strategy in Ethiopia.Resource Tracking and Management Project. Boston, Massachusetts and Addis Ababa, Ethiopia; 2015.https://fpfinancingroadmap.org/data/files/resource-documents/hcfs_review_final_report_september_2015_0.pdf. [Last accessed on 2022 Feb 04]

[13] Transitional Government of Ethiopia. Health Policy of the Transitional Government of Ethiopia. Addis Ababa; 1993. https://www.mindbank.info/item/721. [Last accessed on 2021 Dec 2].

[14] FMoH. Health Care Financing Strategy 2017 – 2025. Addis Ababa, Ethiopia; 2017. Report No.: 2.

[15] FMoH. Annual Health Sector Performance Report 211EFY(2018/19). Addis Ababa, Ethiopia; 2018. https://www.thepalladiumgroup.com/downloads/c442edbcdc749cd6dba609ada08a257b. [Last accessed on 2021 Dec 2].

[16] FDRE. Social Health Insurance proclamation No.690/2010. Addis Ababa: 16th ye ar No.50. 19th August 2010. Federal Negarit Gazeta of the Federal Democratic Republic of Ethiopia. 2010. https://www.ilo.org/dyn/travail/docs/269/Social%20Health%20Insurance%20Proclamation%202010.pdf. [Last accessed on 2021 April 11].

[17] Federal Democratic Republic of Ethiopia. Social Health Insurance scheme Council of Ministers Regulation No. 271/2012. Addis Ababa: 19th year No.3. Addis Ababa: Federal Negarit Gazeta of the Federal Democratic Republic of Ethiopia; 2012. http://ilo.org/dyn/natlex/natlex4.detail?p_lang=en&p_isn=89591&p_country=ETH&p_count=141&p_classification=15&p_classcount=5. [Last accessed on 2021 Dec 11].

[18] Federal Democratic Republic of Ethiopia. Ethiopian Health Insurance Agency establishment Council of Ministers Regulation No. 191/2010. Addis Ababa; 2010. https://chilot.me/wp-content/uploads/2011/12/reg-no-191-2010-the-ethiopian-health-insurance-agency.pdf. [Last accessed on 2021 Dec 11].

[19] EFMoH. Health Sector Transformation Plan (2015/16–2019/20). Vol 20. Addis Ababa, Ethiopia; 2015. https://www.globalfinancingfacility.org/ethiopia-health-sector-transformation-plan-201516-201920. [Last accessed on 2021 Dec 2].

[20] Lavers T (2019). Towards Universal Health Coverage in Ethiopia's ‘developmental state’? The political drivers of health insurance. Soc Sci Med.

[21] Tadele W, Aklilu M, Getaneh Y, Defar A, Acham Y (2015). Willingness to pay for social health insurance among staffs in Ethiopian Public Health Institute, Addis Ababa. Ethiop j public Heal nutr.

[22] Gidey MT, Gebretekle GB, Hogan ME, Fenta TG. Willingness to pay for social health insurance and its determinants among public servants in Mekelle City , Northern Ethiopia : a mixed methods study. Cost Eff Resour Alloc. 2019;17(2)1–12. Available at: 10.1186/s12962-019-0171-x 10.1186/s12962-019-0171-x PMC6332701 30675133

[23] Obse A, Ryan M, Heidenreich S, Normand C, Hailemariam D (2016). Eliciting preferences for social health insurance in Ethiopia: A discrete choice experiment. Health Policy Plan.

[24] United Nations - World Population Prospects. Addis Ababa, Ethiopia Metro Area Population 1950–2021. https://worldpopulationreview.com/en/world-cities/addis-ababa-population. [Last accessed on 2021 Dec 2].

[25] Addis Ababa City adminstration Human resource management and development Bureau Annual report. 2019. http://www.addisababa.gov.et/ar/web/guest/civil-service-agency. [Last accessed on 2021 Dec 26].

[26] Agago TA, Woldie M, Ololo S (2014). Willingness to join and pay for the newly proposed social health insurance among teachers in Wolaita Sodo Town. South Ethiopia Ethiop J Health Sci.

[27] Gidey MT, Gebretekle GB, Hogan ME, Fenta TG (2019). Willingness to pay for social health insurance and its determinants among public servants in Mekelle City, Northern Ethiopia: A mixed methods study. Cost Eff Resour Alloc.

[28] Nosratnejad S, Rashidian A, Mehrara M, Akbari Sari A, Mahdavi G, Moeini M (2014). Willingness to pay for the social health insurance in Iran. Glob J Health Sci.

[29] FMoH. Ethiopian Health Accounts,2016/17. Addis Ababa; 2019. http://www.moh.gov.et/ejcc/sites/default/files/2020.01/Ethiopia%207th%20Health%20Accounts%20Report_2016-17.pdf. [Last accessed on 2021 May 26].

[30] Nguyen LH, Hoang ATD (2017). Willingness to pay for social health insurance in central Vietnam. Front Public Heal.

